# Characterization of a human betaretrovirus in patients with primary biliary cirrhosis

**DOI:** 10.1186/1742-4690-11-S1-O70

**Published:** 2014-01-07

**Authors:** Weiwei Wang, Stan Indik, Shawn T Wasilenko, Gane K-S Wong, Andrew L Mason

**Affiliations:** 1 Department of Medicine, University of Alberta, Edmonton, AB, Canada; 2 Research Institute for Virology and Biomedicine, University of Veterinary Medicine, Vienna, Austria

## 

Our laboratory has characterized and cloned a human betaretrovirus resembling the mouse mammary tumor virus (MMTV) from biliary epithelium and lymph nodes of patients with the autoimmune biliary disease, primary biliary cirrhosis (PBC). Evidence for betaretrovirus infection has been detected mainly in patients’ lymphoid tissue by RT-PCR and immunohistochemistry in approximately 75% of PBC patients. However, the viral burden was observed to be far less in the liver and we could only identify viral sequence in a third of patients using RT-PCR. Other labs have been unable to confirm our data using PCR of hepatic DNA. Not surprisingly, the betaretroviral association with PBC has become a controversial issue. In order to provide more robust evidence of infection in patients with liver disease, we have isolated an infectious betaretrovirus from lymph nodes. We cloned and sequenced the proviral isolates and confirmed viral particles by electron microscopy. We have also conducted ligation-mediated PCR studies with next generation sequencing and found proviral integration sites both in vivo and in vitro. To date, we have identified more than 3,000 integration sites in perihepatic lymph nodes, liver and isolated biliary epithelium in the majority of patients with PBC and seldom in patients with other liver diseases. These data confirm the presence of an MMTV-like virus in patients with autoimmune liver disease. Ongoing studies using biliary disease mouse models with MMTV infection and randomized controlled anti-retroviral trials for patients with PBC will be required to further characterize the association of human betaretrovirus infection with PBC.

